# Optimizing depth and type of high‐throughput sequencing data for microsatellite discovery

**DOI:** 10.1002/aps3.11298

**Published:** 2019-11-03

**Authors:** Mark A. Chapman

**Affiliations:** ^1^ Biological Sciences University of Southampton Life Sciences Building 85, Highfield Campus Southampton SO17 1BJ United Kingdom; ^2^ Centre for Underutilised Crops University of Southampton Life Sciences Building 85, Highfield Campus Southampton SO17 1BJ United Kingdom

**Keywords:** high‐throughput sequencing, marker discovery, microsatellites, simple sequence repeat (SSR) markers

## Abstract

**Premise:**

Simple sequence repeat (SSR) markers (microsatellites) are a mainstay of many labs, especially when working on a limited budget, carrying out preliminary analyses, and in teaching. Whether SSRs mined from plant genomes or transcriptomes are preferred for certain applications, and the depth of sequencing needed to allow efficient SSR discovery, has not been tested.

**Methods:**

I used genome and transcriptome high‐throughput sequencing data at a range of sequencing depths to compare efficacy of SSR identification. I then tested primers from tomato for amplification, polymorphism, and transferability to related species.

**Results:**

Small assemblies (two million read pairs) identified ca. 200–2000 potential markers from the genome assemblies and ca. 600–3650 from the transcriptome assemblies. Genome‐derived contigs were often short, potentially precluding primer design. Genomic SSR primers were less transferable across species but exhibited greater variation (partially explained by being composed of more repeat units) than transcriptome‐derived primers.

**Discussion:**

Small high‐throughput sequencing resources may be sufficient for identification of hundreds of SSRs. Genomic data may be preferable in species with low polymorphism, but transcriptome data may result in longer loci (more amenable to primer design) and primers may be more transferable to related species.

The many advantages of simple sequence repeat (SSR) markers (or microsatellites; reviewed in Hodel et al., 2016a; Vieira et al., 2016) have meant that they continue to be widely used in a variety of biological disciplines, including forensics, paternity testing, population genetics, genetic mapping, and phylogeography. Despite new high‐throughput sequencing (HTS) approaches being able to “genotype by sequence” thousands of markers (single‐nucleotide polymorphisms [SNPs]) in any number of individuals in a short timeframe and at low cost per datapoint, SSRs remain a cost‐effective and informative marker system (reviewed in Hodel et al., 2016a). For example, genotype‐by‐sequencing approaches are only cost‐effective when the number of individuals analyzed is relatively large (and usually in multiples of ca. 96 to aid automated library prep) and are therefore not always appropriate for preliminary investigations, or when budget is limited (including, for example, undergraduate projects).

Traditional isolation of SSR markers through enriched libraries has been largely superseded by the ability to “mine” HTS data from a single individual of a species and to identify SSRs in silico. This approach needs to be followed up by lab analysis to ensure the markers are reproducible and polymorphic, but generally yields a minimum of dozens of microsatellite markers from a single individual. Short‐read data (50–300‐bp reads, e.g., Illumina HiSeq and MiSeq) are by far the most common HTS data and generally require assembly into (longer) loci before identifying SSRs. However, long‐read data (ca. 400‐bp reads [Roche 454, now discontinued] to 50+ kbp reads [Pacific Biosciences and Oxford Nanopore]), where available, can be used for SSR mining without the need for assembly (e.g., Castoe et al., 2010; Wöhrmann et al., 2016). Mining for SSRs can utilize either transcriptome HTS data (e.g., Twyford et al., 2013; Li and Zhang, 2015; Wöhrmann et al., 2016) or genome HTS data (e.g., Owusu et al., 2013; Nock et al., 2014; Bentley et al., 2015), but the efficacy of these two sources has not been compared for plants.

There are several reasons why either transcriptomic or genomic sources might be preferred over the other, depending on the goal of the particular study. Transcriptome‐derived SSRs are, by definition, linked to a transcribed locus, and therefore it might be that these loci are influenced by selection (Morgante et al., 2002; however, neutrality is rarely tested). In some cases this can be an advantage, for example, if the goal is to identify candidate genes with evidence for selection (e.g., Vigouroux et al., 2002; Chapman et al., 2008). In many cases, proximity of a genomic SSR to a transcribed locus is not tested.

Transcriptome‐derived markers tend to be more conserved at the sequence level, which means the markers usually show less polymorphism than random genome‐wide SSR markers (Chabane et al., 2005; Pashley et al., 2006) but are more transferable between species (Liewlaksaneeyanawin et al., 2004; Varshney et al., 2005; reviewed in Ellis and Burke, 2007). The ease of identification and ubiquity of SSRs in plant transcriptomes are highlighted by a recent in silico analysis of transcriptomes from more than 1000 plant species, which revealed over 5.7 million putative SSR markers (Hodel et al., 2016b).

Unless the relevant data are already available, the largest cost for undertaking a study that uses HTS to identify SSRs will likely be the cost of the initial data. It is therefore pertinent that the amount of sequence data generated is not over‐ or underestimated (resulting in a study that is more expensive than necessary or resulting in so few SSRs that the final goal of the study is not achievable, respectively). This opens the question of how much data is sufficient to derive sufficient microsatellite markers for the required application (from a dozen for population genetics to several hundred or more for a genome‐wide association analysis or linkage mapping). In this study I tested this, using a range of input HTS read numbers from five plant species and comparing the outcomes for genome‐ and transcriptome‐derived HTS data. I show that the identification of a few dozen SSR loci can, for the most part, be achieved through assembling very small HTS data sets. In addition, I show that marker transferability between species can be relatively high; therefore, HTS resources from a related species have the potential to be utilized to develop markers in a species without resources. This increases the efficiency of SSR identification in non‐model species; however, transferability in different groups of species is likely to be variable.

## METHODS

### Data and read trimming

Illumina‐derived HTS data are currently the most ubiquitous in public repositories, including the National Center for Biotechnology Information (NCBI) Short Read Archive (SRA; https://www.ncbi.nlm.nih.gov/sra), and hence I focused on this. Data from five species were used; four are model species (*Arabidopsis* Heynh. [*Arabidopsis thaliana* (L.) Heynh.], rice [*Oryza sativa* L.], soybean [*Glycine max* (L.) Merr.], and tomato [*Solanum lycopersicum* L.]), and the fifth is an underutilized legume with a recently sequenced genome (lablab, *Lablab purpureus* (L.) Sweet; Fabaceae; Chang et al., 2018). The species span a range of genome sizes (157–1078 Mbp/1C). The SRA was searched to identify genome and transcriptome HTS data that came from the same accession (Appendix S1). Files were selected at random, with the only stipulations being that each had >30 million (M) paired‐end reads and that reads were between 100 and 150 bp. The following samples were used (with the transcriptome tissues indicated): *Arabidopsis* ecotype Landsberg *erecta* (seedlings), lablab cv. Highworth (sepal); rice cv. Nipponbare (roots), soybean cv. Williams 82 (shoot apical meristem), and tomato cv. M82 (stems).

Trimmomatic (Bolger et al., 2014) was used to identify and remove low‐quality reads using the settings LEADING:5 TRAILING:5 SLIDINGWINDOW:4:15 MINLEN:72. The read files were then parsed down to four different library sizes; 2M, 5M, 10M, 20M. For the lablab transcriptome data, the Trimmomatic step removed a large number of reads, hence the largest library size was 19.07M reads. A resource of 2M reads represents a very small resource, and this depth of sequencing would allow 10–12 samples to be multiplexed on a single MiSeq lane, and up to 100 on a lane of HiSeq.

### Genome assemblies

The 20 pairs of genome‐derived reads (five species × four library sizes) were each assembled using ABySS (ver. 1.3.6; Simpson et al., 2009). Two different *k*‐mer sizes were used (64 and 56). Assembly statistics were taken from the ABySS output, and BUSCO (Benchmarking Universal Single‐Copy Orthologs; Simao et al., 2015) was used to provide a metric for genome assembly completeness. No further attempt to improve the assemblies was made because the goal of this work is to propose simple methods to identify SSRs rather than to optimize genome assembly (reviewed elsewhere; e.g., Wences and Schatz, 2015).

### Transcriptome assemblies

The 20 pairs of transcriptome‐derived reads (five species × four library sizes) were each assembled using Trinity (ver. 2.4.0; Haas et al., 2013). Initially, the reads were normalized to reduce the presence of over‐represented *k*‐mers. Assembly followed the approach of Chapman (2015). Assembly statistics were calculated using the Trinity script *TrinityStats.pl*. N50 is not a valid indicator of transcriptome quality because longer contigs do not necessarily imply a better assembly; therefore, the E90N50 method was used (the N50 based on the 90% most highly expressed transcripts). This was carried out by mapping the initial reads (not normalized) to the transcriptome using the script *align_and_estimate_abundance.pl* in Trinity and RSEM (Li and Dewey, 2011), and then using the Trinity script *contig_ExN50_statistic.pl*.

### SSR identification

Initially, short contigs (<500 bp) were removed from each of the assemblies in order to expedite the SSR identification. Although this step is optional, this should enrich for loci where there is a greater chance of successful primer design because short loci are more likely to have the SSR in or near the end of the fragment (preventing or reducing the chance of successful primer design). However, the genome assemblies based on small numbers of reads only contained a very few contigs longer than 500 bp; therefore, SSRs were identified in the complete assemblies as well as for only the contigs longer than 500 bp.

SSRs were identified using MISA (Thiel et al., 2003; http://pgrc.ipk-gatersleben.de/misa/) with the following minimum number of uninterrupted repeats: eight for dinucleotides, six for trinucleotides, and four for tetranucleotides. SSRs were also identified in the complete genome sequences (the four model species’ genomes were downloaded from https://phytozome.jgi.doe.gov/pz/portal.html and the lablab genome was downloaded from https://db.cngb.org/ [accession CNA0000021]) for comparison.

### Tomato in silico primer design and amplification

Primers were designed to amplify SSRs from the 20M read genome and transcriptome assemblies for tomato. The *k*‐mer = 56 genome assembly was used because of its slightly higher N50 and more total bases than the *k*‐mer = 64 assembly. Only contigs longer than 500 bp were analyzed, and if the SSR identified was in the first or last 50 bp (and therefore would have made primer design more difficult) this locus was removed. Trinity assembles multiple transcripts into genes, and therefore only one transcript per gene (the one with the longest SSR) was retained. If more than one SSR was present in a locus then only the longest was focused on. Because longer SSRs tend to be more polymorphic, I then ranked the loci by SSR length and designed primers to flank the six longest di‐, tri‐, and tetranucleotide SSRs from both the genome and transcriptome (total 36 primer pairs). Primers were designed with batchprimer3 (default settings, except amplicon length was limited to 120–400 bp, and primer length was min 18, max 24, opt 20). If primers could not be designed for one of the “top six” loci, the next on the list was used. The primer sequences were tested in silico as well as in the lab by using PCR.

For the in silico analysis, primer pairs were examined for potential amplification in the genome sequences of the wild tomato species *S. pimpinellifolium* L. (the progenitor species of cultivated tomato) and *S. pennellii* Correll (a more distant relative), both of which were downloaded from ftp://ftp.solgenomics.net/genomes/ (accessions LA0480 and LA0716, respectively). The former is relatively fragmented, and therefore contigs less than 200 bp were removed. The latter is scaffolded to the chromosome level. FastPCR (Kalendar et al., 2017) was used to search the genome sequences using the 36 primer pairs described above as the query sequences. Only hits for which the respective pair was within 5 kbp were considered, and the minimum percentage match between the primer and the genome sequence was set to 85%. The number of hits was counted, as well as the greatest average percentage match between the primers and the genome, both of which could be instructive when considering cross‐species amplification.

To test for amplification success, polymorphism, and transferability across species, DNA was extracted from eight accessions of *Solanum lycopersicum* (including four var. *cerasiforme* (Alef.) Voss), four of *S. pimpinellifolium*, and two accessions of each *S. cheesmaniae* (L. Riley) Fosberg, *S. chmielewskii* (C. M. Rick, Kesicki, Fobes & M. Holle) D. M. Spooner, G. J. Anderson & R. K. Jansen, *S. galapagense* S. C. Darwin & Peralta, *S. habrochaites* S. Knapp & D. M. Spooner, and *S. pennellii*, using a modified Doyle and Doyle (1990) cetyltrimethylammonium bromide (CTAB)–based procedure (see Chapman et al., 2008). Seeds were obtained from the Tomato Genetics Resource Center at University of California, Davis (Appendix S2). Prior to germination, seeds were soaked in 50% commercial bleach for 30 min, rinsed several times, and then germinated on damp filter paper. After germination, seedlings were transferred to 3 : 1 compost : vermiculite in a growth room with 16‐h photoperiod and 23°C day : 18°C night temperature. DNA was extracted from young plants, one per accession.

A universal M13 sequence (CACGACGTTGTAAAACGAC) was appended to the 5′ end of the forward primers to allow a labeled (FAM, TET, or NED) primer with the M13 sequence to be incorporated during PCR (Schuelke, 2000). Initially, amplification success was judged for the eight *S. lycopersicum* and four *S. pimpinellifolium* samples. PCR followed standard procedures with a single annealing temperature of 55°C for all primer pairs, as detailed in Chapman et al. (2008). Amplification success was determined on 1.2% agarose gels. Where successful amplification was identified (>50% of samples and amplicon <400 bp), the samples were sent for genotyping on an ABI PRISM 3730xl (Applied Biosystems, Foster City, California, USA) in the University of Oxford, Department of Zoology, with the GeneScan 500 size standard. Loci were combined such that 3–4 differently labeled and sized loci could be resolved in each lane. For primers that produced a clear and relatively easy‐to‐score product, amplification was tested on the other species.

Amplicon sizes were estimated using GeneMarker ver. 2.4.0 (SoftGenetics, State College, Pennsylvania, USA), and measures of polymorphism (number of alleles, observed and expected heterozygosity) were calculated per locus using GenAlEx ver. 6.5 (Peakall and Smouse, 2006). Polymorphism information content (PIC) was calculated in CERVUS (Kalinowski et al., 2007). Principal coordinate analysis (PCoA; also in GenAlEx) was conducted to investigate the relationships among samples.

## RESULTS

### Genome assemblies

**Figure 1:**
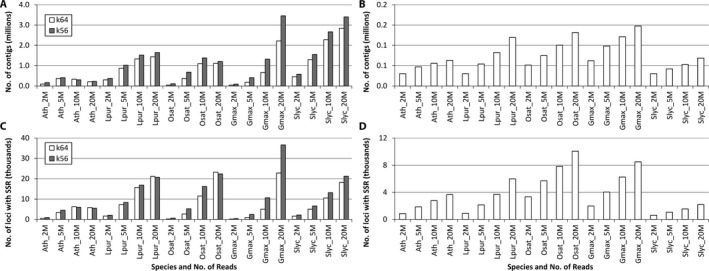
Number of contigs (A, B) and number of loci with simple sequence repeats (C, D) derived from the genome (A, C) and transcriptome (B, D) assemblies. Two *k*‐mer sizes (k56 and k64) were used in the genome assemblies. Species analyzed are abbreviated as follows: Ath = *Arabidopsis thaliana* (*Arabidopsis*); Lpur = *Lablab purpureus* (lablab); Osat = *Oryza sativa* (rice); Gmax = *Glycine max* (soybean); Slyc = *Solanum lycopersicum* (tomato). Four different depths of sequencing were used (2M, 5M, 10M, and 20M reads). Further metrics are given in Appendices S1 and S3.

The results for the two different *k*‐mer settings were almost identical, hence only the results for *k*‐mer = 64 are discussed, but the data for both are presented (Appendices S1, S3; Fig. 1).

**Figure 2:**
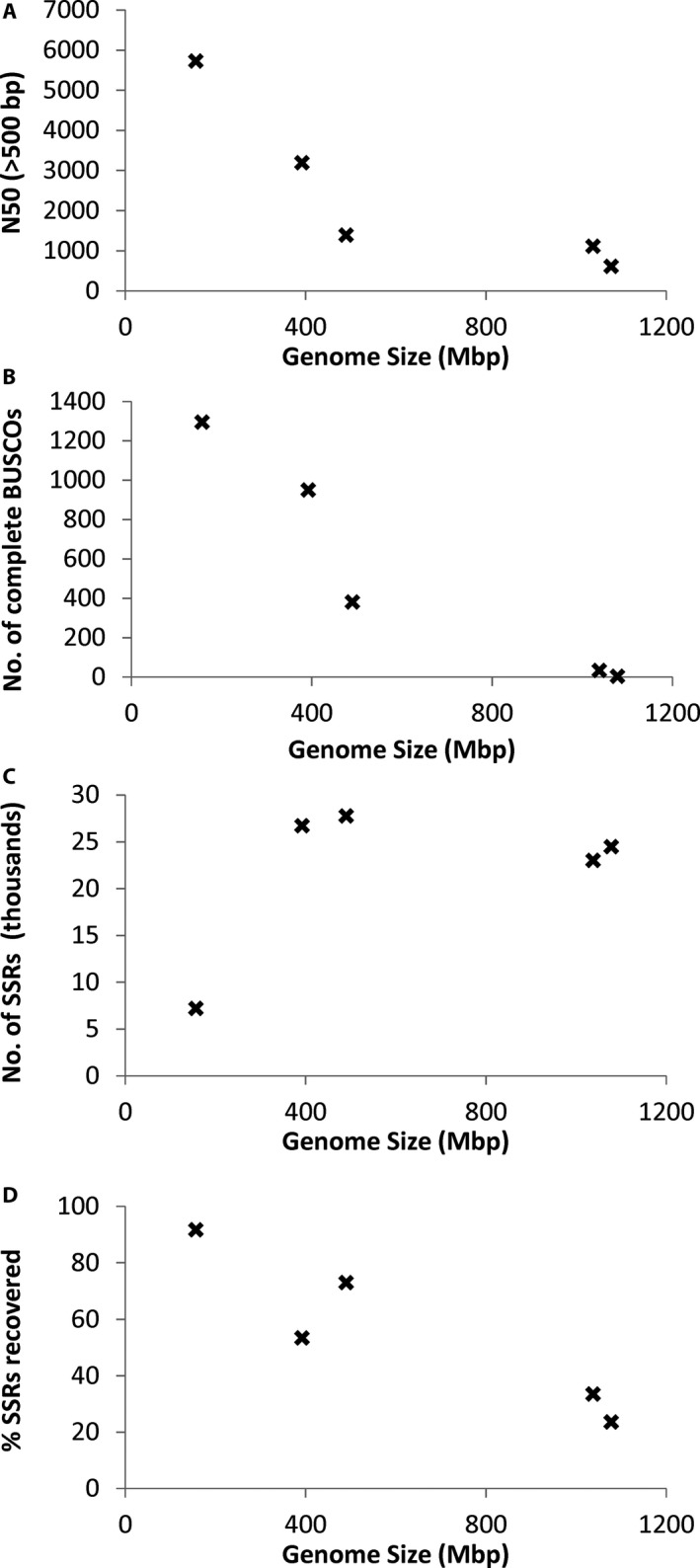
Correlations between genome size and N50 (A), number of complete BUSCOs (Benchmarking Universal Single‐Copy Orthologs) (B), number of simple sequence repeat (SSR) markers identified (C), and the percentage of SSRs recovered (i.e., relative to the number of SSRs predicted from the published genome sequence) (D).

Except for *Arabidopsis*, greater read numbers resulted in longer assemblies and more contigs (Appendices S1, S3; Fig 1A). For *Arabidopsis*, which has the smallest genome of the species investigated, the 20M read assemblies showed a similar total assembly length and a slight decrease in number of contigs relative to the 10M read assemblies, but also demonstrated a considerable increase in the N50 (Appendices S1, S3). For the other taxa, the N50 was also greatest for the 20M read assemblies, with the exception of soybean (Appendices S1, S3). N50 was negatively but not significantly correlated with genome size (ρ = −0.861, *P* = 0.061; Fig. 2A). Length of the longest assembly from each species suggests that at best between 30.4% (soybean) and 103.0% (lablab) of the genome is covered (Appendix S1). The BUSCO analysis suggests the *Arabidopsis* assembly contains 90.0% complete and single‐copy BUSCOs (1294/1440), whereas for soybean this value was only 0.1% (2/1440) (Appendix S4). There was a significant negative correlation between genome size and number of complete single‐copy BUSCOs (ρ = −0.942, *P* = 0.017; Fig. 2B).

Because efficient primer design requires sufficient flanking regions, I also noted the number of contigs greater than 500 bp in all assemblies. For *Arabidopsis* and lablab, the species with the smallest genomes (157 and 392 Mbp, respectively), there were more contigs greater than 500 bp in the assembly based on 10M reads than with 20M reads (Appendices S1, S3), likely because of the considerably greater N50 with 20M reads. For the other three species, the greatest number of contigs longer than 500 bp was found for the 20M read assemblies. For soybean this number was much lower than for rice and tomato (4972 vs. 169,458 and 369,149, respectively), despite the total number of contigs overall being intermediate to the other two (ca. 2.2M vs. 1.1M and 2.8M, respectively). Soybean does have the largest genome of the species under investigation (1078 Mbp), but only marginally larger than tomato (1038 Mbp).

### Transcriptome assemblies

For all species, assembling more reads resulted in an overall larger transcriptome, more contigs, more contigs longer than 500 bp, and a greater N50 and E90N50 (Appendices S1, S3; Fig. 1B). Total assembly size and the number of transcripts assembled (with 20M reads) ranged only 2.5‐fold across species, from 69.3 Mbp to 145.5 Mbp in total length and 63,000 to 147,000 contigs, respectively (Appendices S1, S3), which is not as pronounced as for the genome assemblies. This might be expected because of the seven‐fold different genome sizes of these species, and less than three‐fold difference in gene content (20,946 in lablab [Chang et al., 2018] to 56,044 in *Glycine* [https://phytozome.jgi.doe.gov/]). The number of transcripts in each transcriptome is also affected by the complexity of the tissue being sequenced (Schmid et al., 2005), and each sample here was from a different tissue.

N50 and E90N50 both also increase when assembling greater numbers of reads (Appendices S1, S3), indicating assembly of more long contigs. E90N50 values were comparable across species, varying by only 30% (e.g., range 1429–1839 for the 20M read assemblies).

### SSR identification

**Figure 3:**
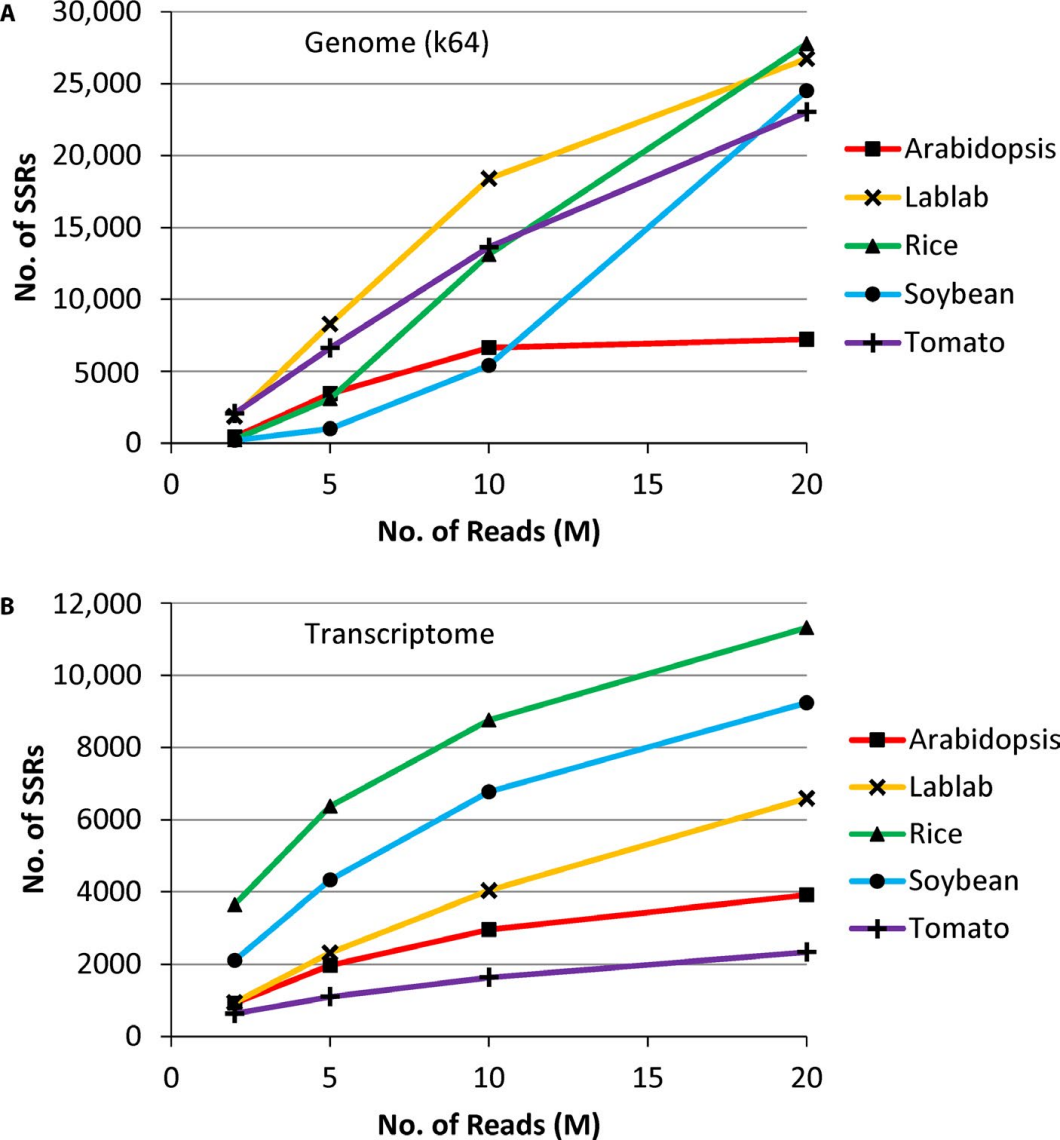
Simple sequence repeat (SSR) discovery with increasing input data based on genome (A) and transcriptome (B) high‐throughput sequencing data. M = millions.

The results for the two different *k*‐mer settings were similar for the genome assemblies, and therefore only the results for the *k*‐mer = 64 assemblies are reported here (data from both assemblies are presented in Appendices S1, S3, and Fig. 1). Predictably, the genome assemblies using more reads, which gave larger assemblies (above), resulted in a greater total number of SSRs predicted and a greater number of loci harboring an SSR (Appendices S1, S3; Figs. 1C, 3A). The number of SSRs ranged from 189 to 2069 with the smallest assemblies (2M reads) and from 7217 to 27,764 for the largest (20M reads). Even smaller assemblies (0.5M reads) resulted in four (*Arabidopsis*) to 347 (tomato) SSRs being identified (data not shown). Considering SSRs present in contigs longer than 500 bp, this number ranged from only four to 85 SSRs in the smallest assemblies (2M reads), highlighting the fragmented nature of these assemblies, and from 240 to 19,939 in the largest assemblies (20M reads) (Appendices S1, S3). For most species, this number was 10–60% lower than in all contigs; however, for soybean this was 99% lower (240 vs. 24,505 SSRs). There was no correlation between genome size and number of SSRs identified (ρ = 0.482, *P* = 0.411; Fig. 2C).

As described above, there was little improvement between the 10M and 20M read genome assemblies for *Arabidopsis*, with only a slight increase in the number of SSRs identified. This is in line with the assembly statistics (above), which demonstrated that there was little increase in the percentage of the genome covered by the 20M assembly over the 10M assembly. Comparing the number of SSRs identified in all contigs in the 20M assembly (7217) with the number of SSRs predicted from the published genome sequence (7875), this implies ca. 91.6% of SSRs have been identified (although this also implies that the 20M assembly has resolved exactly the same loci as the whole genome sequence). This value was lower for the other assemblies, being 53.5%, 73.1%, 23.7%, and 33.5% for the lablab, rice, soybean, and tomato 20M assemblies. There was a significant negative correlation between the percentage of SSRs recovered and genome size (ρ = −0.928, *P* = 0.023; Fig. 2D).

Increasing the input data size for the transcriptome assemblies resulted in an increase in the number of SSRs identified (Figs. 1D, 3B); this was highest for rice (11,317 SSRs in contigs >500 bp) and lowest for tomato (2337 SSRs in contigs >500 bp) in the 20M read assemblies. Overall, more SSRs were identified in the genome assemblies than the transcriptome assemblies (7217–27,764 per species vs. 2337–11,317; Appendices S1, S3); however, the percentage of loci with an SSR was less in the genome assemblies than for the transcriptome assemblies (0.6–2.8% vs. 3.2–7.6%; Appendices S1, S3).

Dinucleotide repeat SSRs were the most prevalent in the larger assemblies of the genomes of all species except tomato, where trinucleotide repeat SSRs prevailed (Appendix S5). The smallest assembly for *Arabidopsis* consisted of more tri‐ than dinucleotide repeats, and the two smallest assemblies (2M and 5M reads) for lablab contained more tetra‐ than di‐ or trinucleotide repeats (Appendix S5). Trinucleotide repeat SSRs were the most common for all assembly sizes for the transcriptomes of *Arabidopsis*, rice, soybean, and tomato, but dinucleotide repeats were more common in the lablab transcriptome (Appendix S5).

### In silico and empirical SSR amplification success from in silico–designed SSR primers

For primer design, SSR‐containing loci from the tomato genome and transcriptome (20M reads) assemblies were reduced down to only those loci longer than 500 bp, which resulted in 7361 loci (containing 9184 SSRs) for the genome assembly and 2190 transcripts (containing 2337 SSRs) for the transcriptome assembly. After removing subsets of loci to expedite SSR identification (see Methods), there remained 5583 and 681 SSR‐containing loci from the genome and transcriptome assemblies, respectively. From these, primers were designed to span the six longest SSRs of each repeat type (di‐, tri‐, and tetranucleotide). In the genome assembly, primers could not be designed for one of the “top six” tetranucleotide loci; therefore, the seventh was selected. Similarly, in the transcriptome assembly, one each of the di‐ and trinucleotide repeat loci failed primer design, and the next on the list was used. Primer details are given in Appendix S2.

In the in silico study, 16 of the 18 (89%) genomic SSR primer pairs and 13 of the 18 (72%) transcriptomic SSR primer pairs had at least one potential match in the *S. pimpinellifolium* genome, with 10 (56%) and seven (39%), respectively, having at least one potential match in the *S. pennellii* genome (Appendix S2). The average number of potential hits in the genome for the genomic SSRs was more than twice that of the transcriptomic SSRs (2.38 vs. 1.00 in *S. pimpinellifolium* and 2.80 vs. 1.30 in *S. pennellii*; Appendices S2, S6A, B). In addition, the maximum percentage identity between the primers and the genome was significantly greater for the transcriptomic SSR primers than the genomic SSRs in *S. pennellii* (Mann–Whitney *U*‐test, W = 63.50, *P* = 0.011) but not in *S. pimpinellifolium* (Mann–Whitney *U*‐test, W = 243.00, *P* = 0.913).

In the PCR assay, 14 of the 18 (78%) genomic SSR primer pairs and 11 of the 18 (61%) transcriptomic SSR primer pairs generated an amplicon in at least six of the 12 *S. lycopersicum* and *S. pimpinellifolium* samples (Appendix S2). One of the primer pairs for a transcriptomic SSR produced an amplicon larger than anticipated (ca. 700 bp) and so was not considered further. The average number of successful amplifications in *S. lycopersicum* and *S. pimpinellifolium* was 11.4 ± 0.9 (mean ± SD) for the genomic SSR primers and 9.7 ± 2.2 for the transcriptomic SSRs. Despite these markers being developed from the longest SSR loci in the assemblies, none exhibited extensive stutter. Polymorphism (Appendix S2) in the 12 *S. lycopersicum* and *S. pimpinellifolium* samples was significantly greater for genomic than transcriptomic SSRs, based on number of alleles (*A*; 5.8 ± 1.8 vs. 3.5 ± 1.8; *t*‐test: *t* = 3.05, *P* = 0.007; Appendix S6C), expected heterozygosity (*H*_e_; 0.71 ± 0.10 vs. 0.50 ± 0.27; *t*‐test: *t* = 2.28, *P* = 0.045), and PIC (0.67 ± 0.13 vs. 0.46 ± 0.26; *t*‐test: *t* = 2.31, *P* = 0.041). There was a significant correlation between the number of repeats present in the reference allele and *A*,* H*_e_, and PIC, conforming to the expectation that longer SSRs tend to be more polymorphic. The difference in polymorphism between genomic and transcriptomic SSRs is therefore likely to be, at least in part, due to the length of the underlying reference allele.

The PCoA plot (Appendix S7) demonstrates genetic differentiation between *S. lycopersicum* and *S. pimpinellifolium*, as predicted.

The 24 primers (14 genomic and 10 transcriptomic; i.e., excluding the 700‐bp locus) were also tested in a range of other wild tomato species to determine the likelihood that markers can be used across other species. This transferability was significantly greater for the transcriptome‐derived SSR primers than for the genomic SSR primers (average [out of 10] = 9.40 ± 1.90 vs. 6.71 ± 3.67; *t*‐test: *t* = 2.34, *P* = 0.030; Appendix S2). At least nine out of the 10 transcriptomic SSR primer pairs amplified in both individuals of each species analyzed, whereas between 50% and 86% of the 14 genomic SSR primer pairs amplified in both individuals of each species (Appendix S6D).

## DISCUSSION

The goal of this investigation was to assess sequencing depth (a 10‐fold range of input read numbers) on the ability to assemble an HTS resource for the purpose of identifying SSR loci in plants. If resources are already available, this can be exploited (in some cases from a related species, but see considerations below); when resources are not available, it is becoming routine to generate these as needed. For the purposes of identifying SSR loci, it is important to understand the depth of sequencing needed in order to make SSR discovery cost‐effective, as well as the extent to which SSR loci might be transferable across species. To this end, five species were investigated at four sequencing depths, and both genome and transcriptome sequences were analyzed. In total, 40 assemblies were generated and analyzed for the presence of SSRs. For tomato, primers were designed and tested both in silico and in the lab for amplification in tomato and a range of wild relatives.

### Depth of sequencing comparison

Although not the focus of this paper, for all species and for both the genome and transcriptome assemblies, more input reads resulted in a longer and more complete overall assembly. For the genome assemblies, more reads resulted in more contigs, except for *Arabidopsis* where fewer but longer contigs (i.e., a greater N50) were assembled, implying a better assembly. N50 and the number of complete single‐copy BUSCOs were negatively correlated with genome size; therefore, species with smaller genomes were exhibiting a better assembly overall than those with large genomes. For the transcriptome assemblies, more reads always resulted in more transcripts, which is as expected because deeper sequencing will uncover more of the lowly expressed transcripts. Mean, median, N50, and E90N50 of the transcriptome assemblies also increased with a greater input number of reads.

The number of SSRs also increased with the size of the input data in the genome assemblies, in line with the assemblies covering more of the genome. There was no correlation between genome size and number of SSRs resolved, which appears to be because approximately the same total number of SSRs were found in four of the five species (with the exception of *Arabidopsis* with the smallest genome); therefore, deeper sequencing (>20M reads) is expected to continue to resolve more SSRs in these species. This is backed up by the observed negative correlation between genome size and the estimated percentage of SSRs in the whole genome that were recovered in the 20M read assemblies.

Because of potential problems associated with designing primers to span SSRs located in short sequences (see Methods), a comparison was made regarding the number of SSRs found in the contigs longer than 500 bp. For the genome assemblies from 2M reads, there were 186 (soybean) to 3693 (lablab) contigs longer than 500 bp, in which only six to 85 SSRs resided. At this sequencing depth, it may be problematic to identify sufficient SSRs in the longer contigs, although reducing the minimum contig size would increase the number of putative SSRs. Reducing the minimum size to 250 bp increased the number of putative SSRs to 15 and 616 in soybean and lablab, respectively.

This low number of identified SSRs was not a result of the assembly being especially suboptimal; for soybean (with the lowest number of SSRs in the 2M read assembly), testing different *k*‐mer settings (24, 48, 56, 64, and 96) only resulted in 31 to 437 contigs longer than 500 bp, and two to 12 SSRs (data not shown).

For soybean, even the largest genome assembly only resulted in 240 putative SSRs in the contigs longer than 500 bp. Soybean has the largest genome, and therefore it could be that 20M genome reads or more are required for SSR identification in species with larger genomes, but 5–10M may suffice for others. Excluding soybean, the 10M read assemblies all identified more than 3000 SSRs in the contigs longer than 500 bp. Based on attrition from the tomato primer design section, where primers could be designed from approximately 75% of loci (i.e., after discarding those with insufficient flanking region) and 14 of the 18 tested amplified and were scorable, this indicates that ca. 58% of in silico–identified genome‐derived SSRs have the potential to be successfully amplified and polymorphic. This would mean that more than 1700 useful SSRs can be identified in the 10M read assemblies of four of the five species. For the fifth species (soybean), this value is only 27 with 10M reads, and therefore the largest (or an even larger) assembly would be preferred.

For the transcriptome assemblies, assembling 2M reads resulted in more than 600 SSRs in the transcripts longer than 500 bp, and for the 20M read assemblies this resulted in more than 2300 SSRs. Using the values for tomato based on the primer design strategy employed (i.e., 69% of loci were discarded due to insufficient flanking region, and 10 of 18 primers amplified a scorable product of expected size), this suggests that the 2M and 20M read assemblies could result in more than 100 and ca. 400 SSR‐containing loci, respectively.

### Genomic vs. transcriptomic comparison

In general, there were more di‐ than tri‐ or tetranucleotide repeat SSR loci in the genome assemblies (with the exception of tomato), and trinucleotide repeat SSR loci were more prevalent in the transcriptome assemblies (with the exception of lablab). This pattern follows previous studies wherein dinucleotide repeats prevail in genomes and trinucleotide repeats are more common in transcriptomes (e.g., in *Glycine*; Hodel et al., 2016b); the latter is presumably because a portion of each transcript is coding, and therefore triplet insertions or deletions will not interrupt the reading frame (Morgante et al., 2002).

At low sequencing depths (2–5M reads), more SSRs were resolved in the transcriptomes of rice and soybean than in the genome, whereas for the highest number of input reads, more SSRs were always resolved in the genome assemblies. There was no clear pattern associated with genome size, and therefore it is more likely that this pattern results from the range of tissues sequenced, which could be more or less complex in their RNA assemblage. Hence it is impossible to conclude whether genomes vs. transcriptomes are better or worse for SSR identification.

However, when it comes to the primer testing, this suggests that the transcriptome primers have the potential to be (1) more reliable (i.e., fewer off‐target amplification) and (2) more transferable across species, but (3) less polymorphic than the genome primers. These properties are key determinants in the utility of these markers for further studies, and therefore could be considered in a future investigation.

Regarding reliability of transcriptome primers, in the in silico analysis, primers designed from the genome assembly had between one and 11 potential hits (2.5 ± 2.9 [mean ± SD]), whereas for the transcriptomic primers there were only one or two potential hits (1.1 ± 0.3). A primer pair that amplifies more than one locus is generally discarded because of the inability to clearly score the alleles, and therefore using transcriptomic SSR primers might be preferred in such cases.

Regarding transferability (and ignoring the potential for multiple amplicons), transferability across species was estimated to be slightly higher in the in silico analysis for the genomic SSRs than the transcriptomic SSRs; however, this was not borne out in the empirical primer testing. When the primers were tested for amplification in other species, significantly more wild tomato DNA samples were amplified with the transcriptome‐derived primers than the genomic primers.

Regarding polymorphism levels, transcriptome‐derived primers had lower polymorphism than the genomic primers in the 12 individuals analyzed (e.g., *A* = 3.5 ± 1.8 vs. 5.8 ± 1.8 and *H*_e_ = 0.50 ± 0.27 vs. 0.71 ± 0.10). In part, this stems from the larger number of repeats in the genome‐derived SSRs than the transcriptome‐derived SSRs.

### Conclusions and recommendations

For species with large genomes, it is likely that assembly of transcriptome HTS reads will result in a higher likelihood of being able to identify SSRs in contigs longer than 500 bp (and hence more likely to permit primer design) than assembling genomic reads, unless a large number of genomic reads are available. It is worth noting that some plant genomes are considerably larger than the largest genomes utilized here (soybean; 1078 Mbp/1C); therefore, for those species with genomes larger than soybean, the assembly of transcriptome HTS reads is encouraged.

Given that a lower number of transcriptome reads was generally required to result in the same number of SSRs as the genome reads, if computational power is limited it might also be prudent to utilize transcriptome reads to the same end.

However, if the target species has low expected polymorphism, such as an endangered species with small population size or a crop with a strong genetic bottleneck, it might be preferred to design SSR primers from genome reads than transcriptome reads simply because polymorphism is likely to be higher from the former. It should be noted, however, that in this work only two of the 10 transcriptomic SSR primers failed to reveal any variation in eight individuals of cultivated tomato, a crop species known to have relatively low genetic diversity (reviewed in Bauche and Causse, 2012); consequently, this is probably a minor concern.

Finally, utilizing HTS resources from a related species is a viable and cost‐effective way to identify polymorphic SSR markers in a species that lacks HTS resources. In this work, I found that the transcriptome‐derived primer pairs were more transferable across species than genome‐derived SSR primer pairs. However, transferability between species can vary greatly (Ellis and Burke, 2007) and is not always a simple function of genetic distance (e.g., Hodel et al., 2016b), and so may have to be tested on a case‐by‐case basis.

## Supporting information


**APPENDIX S1.** Assembly statistics (overall, left) and only contigs longer than 500 bp (right).

**APPENDIX S2.** Details of primers designed; amplicon size; in silico primer amplification (FastPCR); amplification success in tomato (lyc), cherry tomato (lyccer), and its progenitor (pimp); overall statistics for each primer pair; and amplification success in a range of species.

**APPENDIX S3**. Summary statistics derived from the genome (left) and transcriptome (right) assemblies.

**APPENDIX S4.** Summary of the Benchmarking Universal Single‐Copy Orthologs (BUSCO) analysis for the genome assemblies.

**APPENDIX S5.** Relative proportions of di‐, tri‐, and tetranucleotide simple sequence repeats resolved in the genome (*k*‐mer sizes k56 and k64) (A) and transcriptome (B) assemblies.

**APPENDIX S6.** Results of the tomato primer testing. In silico analysis of primer transferability to two wild tomato species, *Solanum pennellii* (A) and *S. pimpinellifolium* (B); number of alleles for the genome‐ and transcriptome‐derived simple sequence repeat (SSR) markers (C); and the transferability of SSR markers across wild tomato species (D).

**APPENDIX S7**. Principal coordinates analysis of tomato (lyc), cherry tomato (lyccer), and wild tomato (pimp) accessions using simple sequence repeat markers identified in this study.

## Data Availability

All raw data were obtained from the National Center for Biotechnology Information (NCBI) Sequence Read Archive; accession numbers are given in the supporting information. Genome sequences were downloaded from the websites cited in the Methods. Scripts used are taken from published software as indicated in the text.
