# Supplementary material for: Optimizing depth and type of high‐throughput sequencing data for microsatellite discovery
Source: Appl Plant Sci. 2019 Nov 3;7(11):e11298. doi: 10.1002/aps3.11298 (PMC6858294; doi:10.1002/aps3.11298)

APPENDIX S3. Summary statistics derived from the genome (left) and transcriptome (right) assemblies. Two *k*-mer sizes (k56 and k64) were used in the genome assemblies. Species analyzed are abbreviated as follows: Ath = *Arabidopsis thaliana* (*Arabidopsis*); Lpur = *Lablab purpureus* (lablab); Osat = *Oryza sativa* (rice); Gmax = *Glycine max* (soybean); Slyc = *Solanum lycopersicum* (tomato). Four different depths of sequencing were used (2M, 5M, 10M, 20M reads).

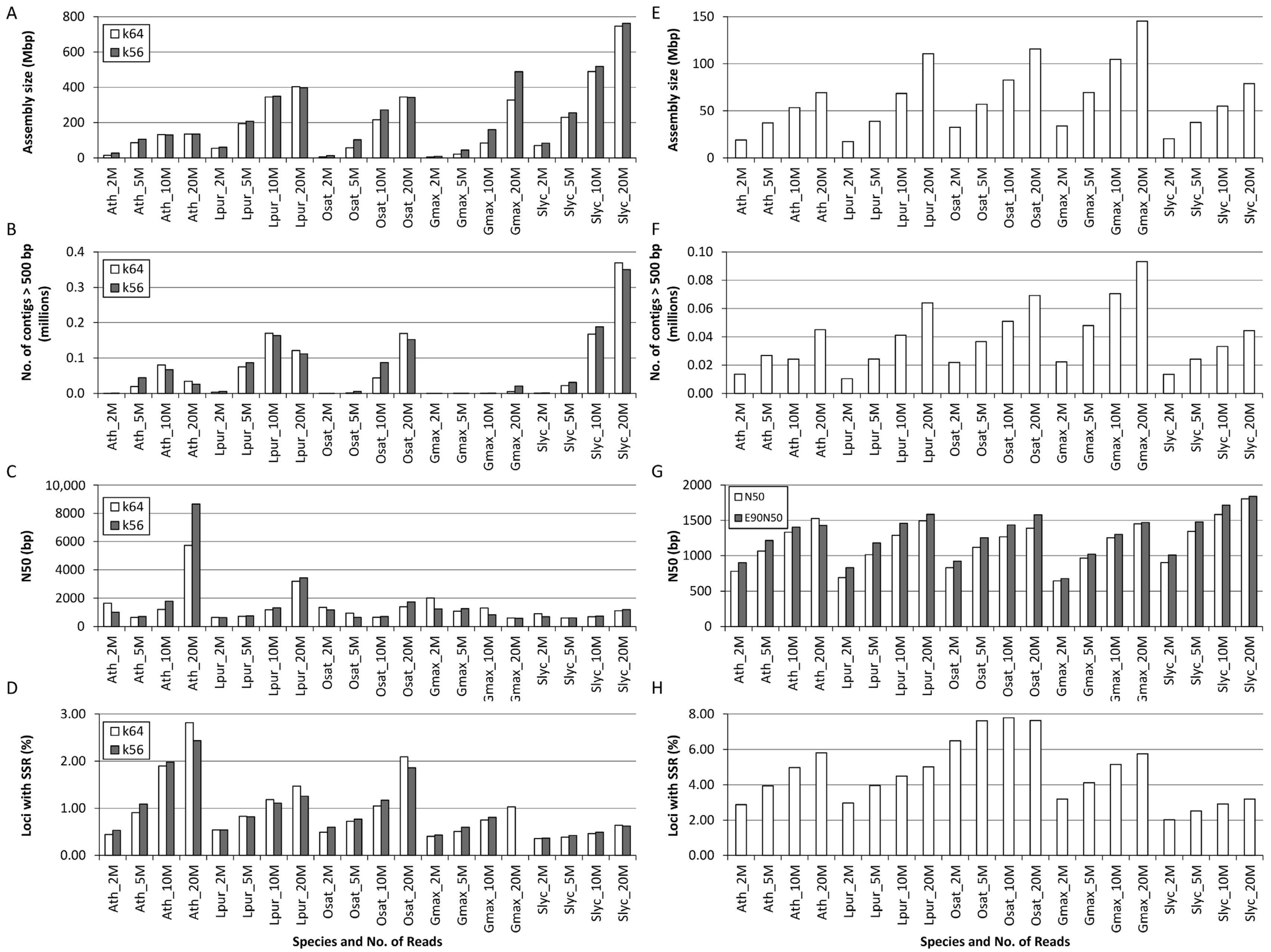

Supplement: Supplementary file 3 — APPENDIX S3. Summary statistics derived from the genome (left) and transcriptome (right) assemblies. [file APS3-7-e11298-s003.pdf]
