# Supplementary material for: Optimizing depth and type of high‐throughput sequencing data for microsatellite discovery
Source: Appl Plant Sci. 2019 Nov 3;7(11):e11298. doi: 10.1002/aps3.11298 (PMC6858294; doi:10.1002/aps3.11298)

DOI

APPENDIX S5. Relative proportions of di-, tri-, and tetranucleotide simple sequence repeats resolved in the genome (*k*-mer sizes k56 and k64) (A) and transcriptome (B) assemblies.

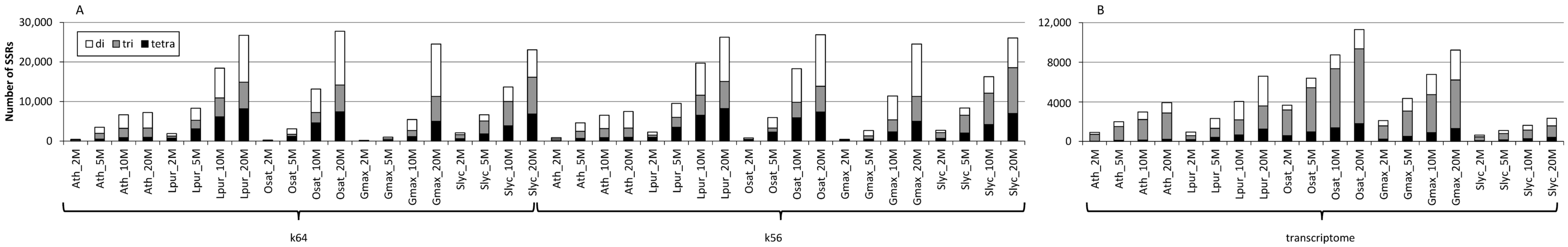

Supplement: Supplementary file 5 — APPENDIX S5. Relative proportions of di‐, tri‐, and tetranucleotide simple sequence repeats resolved in the genome (k‐mer sizes k56 and k64) (A) and transcriptome (B) assemblies. [file APS3-7-e11298-s005.pdf]
