# Supplementary material for: Optimizing depth and type of high‐throughput sequencing data for microsatellite discovery
Source: Appl Plant Sci. 2019 Nov 3;7(11):e11298. doi: 10.1002/aps3.11298 (PMC6858294; doi:10.1002/aps3.11298)

APPENDIX S6. Results of the tomato primer testing. In silico analysis of primer transferability to two wild tomato species, *Solanum pennellii* (A) and *S. pimpinellifolium* (B); number of alleles for the genome- and transcriptome-derived simple sequence repeat (SSR) markers (C); and the transferability of SSR markers across wild tomato species (D).

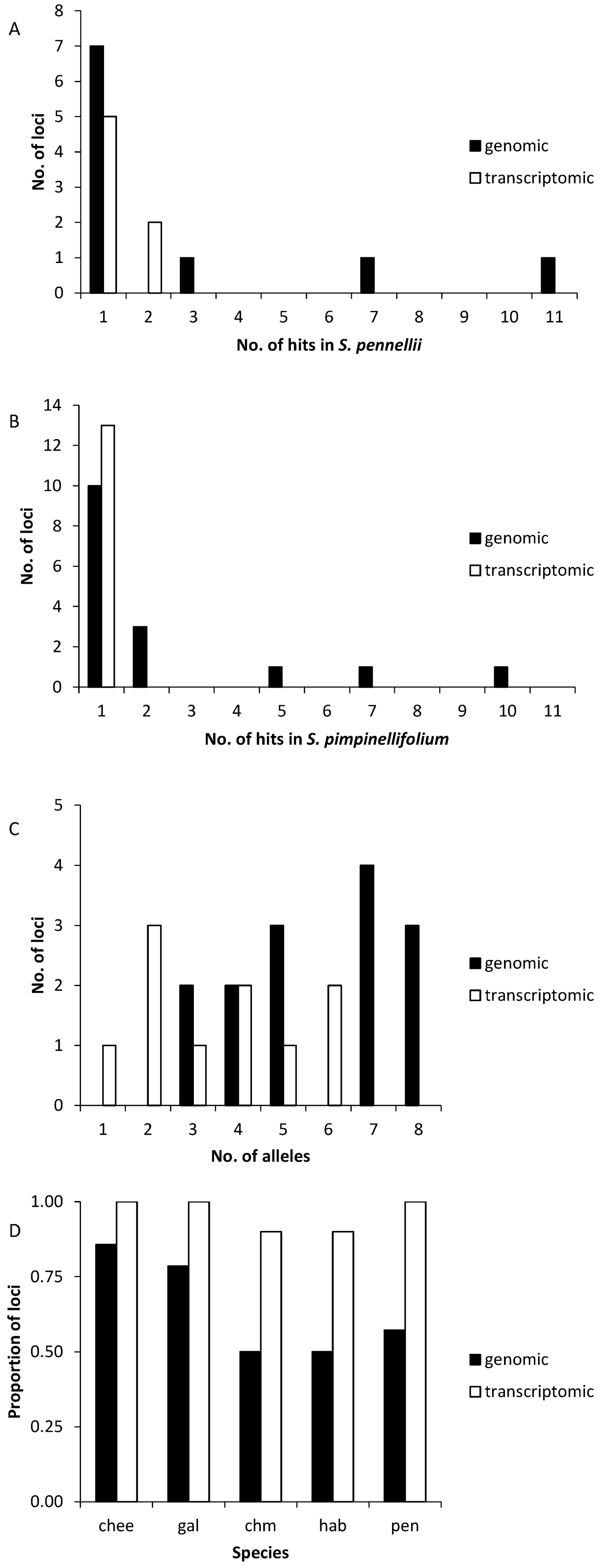

Supplement: Supplementary file 6 — APPENDIX S6. Results of the tomato primer testing. In silico analysis of primer transferability to two wild tomato species, Solanum pennellii (A) and S. pimpinellifolium (B); number of alleles for the genome‐ and transcriptome‐derived simple sequence repeat (SSR) markers (C); and the transferability of SSR markers across wild tomato species (D). [file APS3-7-e11298-s006.pdf]
