# Supplementary material for: Optimizing depth and type of high‐throughput sequencing data for microsatellite discovery
Source: Appl Plant Sci. 2019 Nov 3;7(11):e11298. doi: 10.1002/aps3.11298 (PMC6858294; doi:10.1002/aps3.11298)

APPENDIX S7. Principal coordinates analysis of tomato (lyc), cherry tomato (lyccer), and wild tomato (pimp) accessions using simple sequence repeat markers identified in this study.

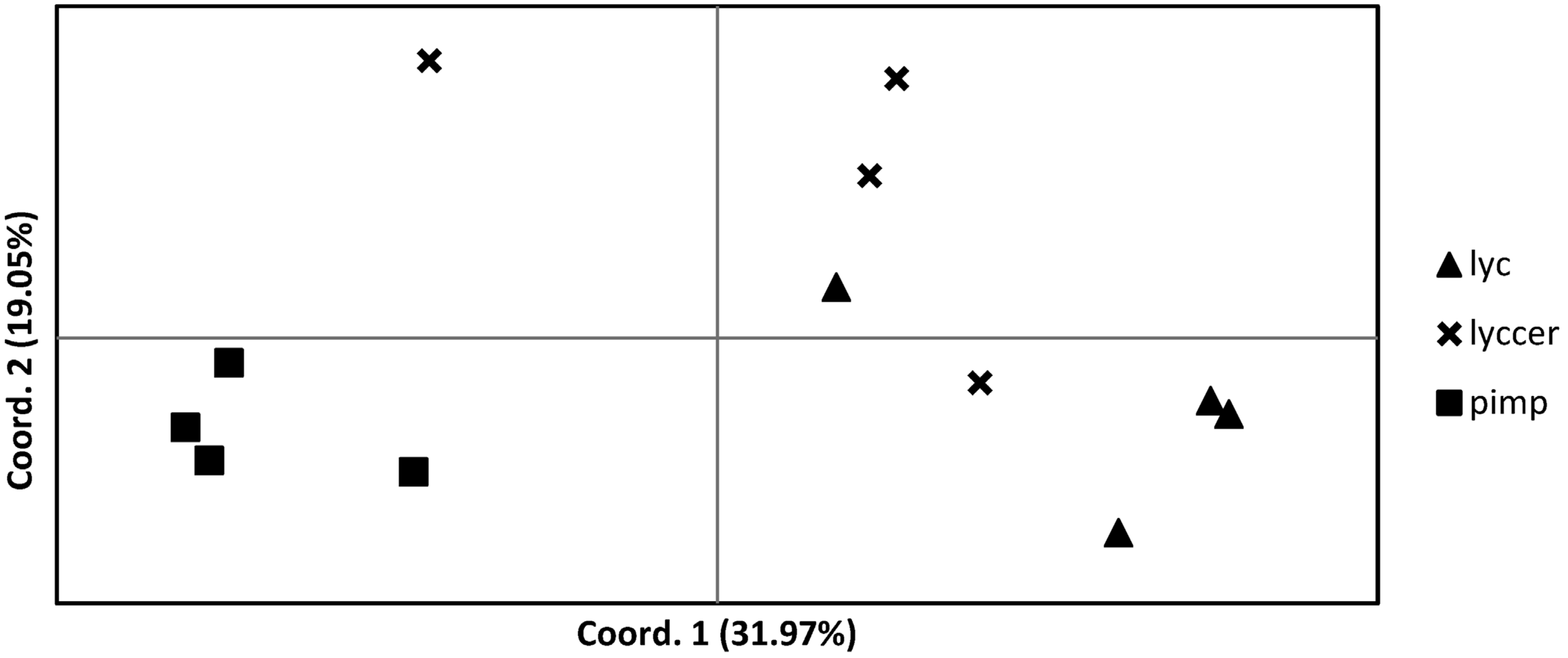

Supplement: Supplementary file 7 — APPENDIX S7. Principal coordinates analysis of tomato (lyc), cherry tomato (lyccer), and wild tomato (pimp) accessions using simple sequence repeat markers identified in this study. [file APS3-7-e11298-s007.pdf]
